# A Label-Free Fluorometric Glutathione Assay Based on a Conformational Switch of G-quadruplex

**DOI:** 10.3390/molecules26092743

**Published:** 2021-05-07

**Authors:** Xi Zhou, Doudou Zhang, Ying Yan, Hailun He, Yukui Zhou, Changbei Ma

**Affiliations:** School of Life Sciences, Central South University, Changsha 410013, China; zhouxi@csu.edu.cn (X.Z.); zddnoe@126.com (D.Z.); Yany2018@csu.edu.cn (Y.Y.); helenhe@csu.edu.cn (H.H.)

**Keywords:** glutathione, G-quadruplex, mercury ions, thioflavin T, fluorescence assay

## Abstract

In this paper, a label-free fluorescent method for glutathione (GSH) detection based on a thioflavin T/G-quadruplex conformational switch is developed. The sensing assay is fabricated depending on the virtue of mercury ions to form a thymine–thymine mismatch, which collapses the distance between two ssDNA and directs the guanine-rich part to form an intra-strand asymmetric split G-quadruplex. The newly formed G-quadruplex efficiently reacts with thioflavin T and enhances the fluorescent intensity. In the presence of GSH, Hg^2+^ is absorbed, destroying the G-quadruplex formation with a significant decrease in fluorescence emission. The proposed fluorescent assay exhibits a linear range between 0.03–5 μM of GSH with a detection limit of 9.8 nM. Furthermore, the efficacy of this method is examined using human serum samples to detect GSH. Besides GSH, other amino acids are also investigated in standard samples, which display satisfactory sensitivity and selectivity. Above all, we develop a method with features including potentiality, facility, sensitivity, and selectivity for analyzing GSH for clinical diagnostics.

## 1. Introduction

Glutathione (GSH), also called γ-glutamyl-cysteinyl-glycine, is a thiol tripeptide containing glutamic acid, cysteine, and glycine [1]. It is the most abundant thiol in human cells and is composed of two characteristic structural elements: a γ-glutamyl linkage and a sulfhydryl group, in which the thiol (-SH) of cysteine residue is the active group [2]. Glutathione plays critical roles in many cellular processes, such as cell growth and metabolism, and, notably, it governs the redox state of organs [3]. In addition, it is a kind of endogenous detoxicant; the active thiol can react with different components, especially heavy metals [1,4,5]. An abnormal glutathione level in organs has been suggested to be tightly connected with various diseases, for example, heart diseases, Alzheimer’s, Parkinson’s, AIDS, cancer, diabetes, and rheumatoid arthritis, and acceleration of the process of aging [3,6]. Owing to its outstanding properties and implications in human diseases, a facile, sensitive, rapid, and time-saving method for the detection of GSH needs to be developed.

Over the past years, different assays to detect glutathione have undergone rapid development, including mass spectrometry (MS), high-performance liquid chromatography (HPLC), electrochemiluminescence, and colorimetry [7,8,9,10,11]. However, the developed methods always exhibited several disadvantages, such as low sensitivity, high cost, and sophisticated instrument manipulation. Recently, fluorescent-based strategies have drawn researchers’ attention, since fluorescence assays offer several advantages, including low cost, high sensitivity, time saving and rapid response. For example, our group designed a fluorescence strategy for detecting glutathione and cysteine based on a Ag+-mediated conformational switch [12]. Tong and her co-workers constructed another fluorescence method for sensing glutathione and cysteine based on conformation-specific G-quadruplex formation induced by ThT [13]. Again, Ma used the features of oxidase-mimicking activity of MnO_2_ nanosheets to developed a dual-sensing method for glutathione and silver (I) [14]. Li and co-workers developed a series of rapid and sensitive methods to detected GSH based on functionalized quantum dots (QDs) and MnO_2_ nanosheets [15,16,17]. A fluorescent nanoprobe was also proposed by Yang Liu to realize GSH, which is dependent on the co-assembly of fluorescein and amphiphilic BODIPY [18]. However, these assays mentioned above always need a complex process, or sensitivity is restrained, so an easier fluorescent strategy that nonetheless has high sensitivity should be developed.

It is well known that the G-quadruplex is formed by G-rich sequences, and it significantly increases the fluorescence intensity when reacting with small molecules, including thioflavin T (ThT), thiazole orange (TO), and N-methyl mesoporphyrin IX (NMM) [19,20,21,22]. ThT is more reliable than other fluorogenic dyes due to its high structural selectivity towards the G-quadruplex. However, selective binding of the DNA bases with metal ions could disrupt G-quadruplex formation, resulting in a decrease in the fluorescence light-up. For example, Hg^2+^ is able to react with thymine–thymine mismatch to form the T-Hg-T structure [23,24,25]. Two groups, Bo Tang and Ruqin Yu, used this mechanism for the detection of biothiols, glutathione, and cysteine. The biothiols can absorb metal ions, which can be used to design label-free fluorescent methods [13,26]. At the same time, Yongxiang Wang reported a technique that uses the T-Hg-T mismatch pairs to close the two split DNA (with G-rich fragments) to form the intra-strand asymmetric split G-quadruplex with a sharp increase in fluorescent intensity [27,28]. Wang’s method is easy to use and has high sensitivity and a low limitation to detect Hg^2+^. Following the above mechanism, we proposed a label-free, stable, highly sensitive, and lower-limitation fluorescent method to detect GSH based on a conformation switch of the G-quadruplex mediated by ThT. Under the optimal parameters, the assay showed a good response to GSH and was successfully detected in human serum samples.

## 2. Results

### 2.1. Strategy of GSH Assay

**Scheme 1:**
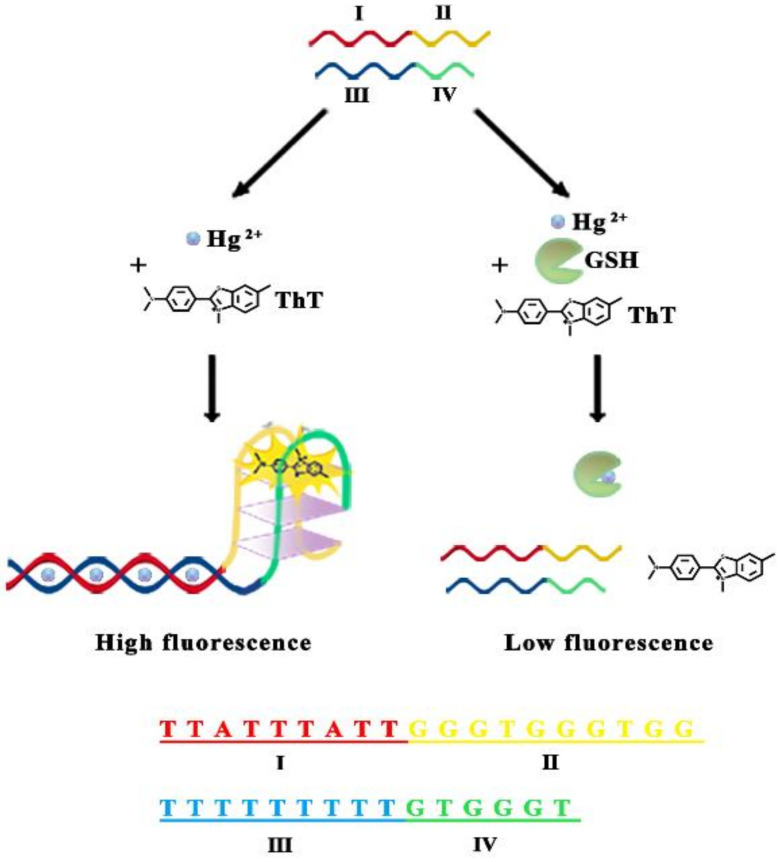
Schematic illustration of the design principle for GSH detection.

The strategy of the proposed fluorescent assay to detect GSH is illustrated in Scheme 1. The system is composed of two G-rich ssDNAs (DNA1 and DNA2), in which DNA1 (domains I and II) and DNA2 (domains III and IV) both include two domains, Hg^2+^ as the T-T mismatched pair mediator, and ThT as the fluorescent probe [27,28]. Domains I and III are T-rich ssDNAs in which the sequences are partially complementary to each other. On the other hand, domains II and IV are G-rich sequences. In the presence of Hg^2+^, the system assembles a mismatched T-Hg-T structure in domains I and III, placing DNA1 and DNA2 close to each other. Consequently, the two G-rich fragments fold into the intra-strand asymmetric split G-quadruplex and then react with ThT for the formation of the split G-quadruplex/ThT complex to spark high fluorescence. The proposed assay caters to a competitive reaction when the target GSH is present in the system. GSH can react with Hg^2+^ and interrupt the formation of T-T-mismatched dsDNA, exhibiting a low fluorescence signal as the outcome. The differences in fluorescence intensity in the presence and absence of the target GSH define the final data; therefore, this strategy can monitor the GSH level in real time.

### 2.2. Feasibility Validation of the Proposed Method

**Figure 1:**
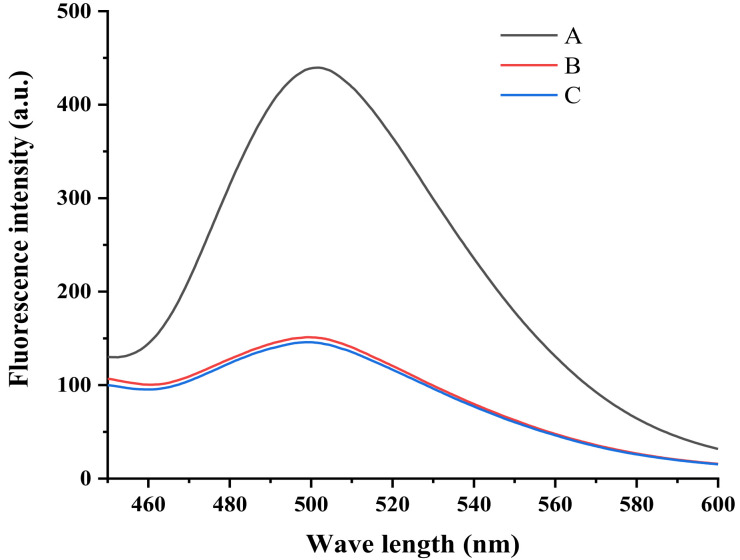
Fluorescence emission spectra under different conditions: (1) DNA1/DNA2+Hg^2+^ (curve A); (2) DNA1/DNA2+Hg^2+^+GSH (curve B); (3) DNA1/DNA2 (curve C).

For the investigation of the feasibility of the assay, different samples were detected by the proposed system. We prepared three kinds of samples: (1) DNA1/DNA2+Hg^2+^ (curve A); (2) DNA1/DNA2+Hg^2+^+GSH (curve B); (3) DNA1/DNA2 (curve C). As shown in Figure 1, when DNA1/DNA2 and Hg^2+^ were added into Tris-HNO_3_ reaction buffer, the fluorescent intensity was found to increase sharply (curve A). As Hg^2+^ acts as a mediator generating T-T mismatch pairs, it assisted the folding of two G-rich fragments into the intra-strand asymmetric split G-quadruplex. Eventually, the newly formed G-quadruplex was compatible to react with ThT. However, when the system was presented with GSH, which could chelate Hg^2+^ to disrupt the rest of the reactions, the fluorescent intensity became weak (curve B). At the same time, the absence of Hg^2+^ and GSH generated deficiency of T-T mismatch pairs, which explains the lessened fluorescent intensity (curve C). Above all, the results show that our method is feasible to detect GSH in real time.

### 2.3. Optimization of the Experimental Conditions

**Figure 2:**
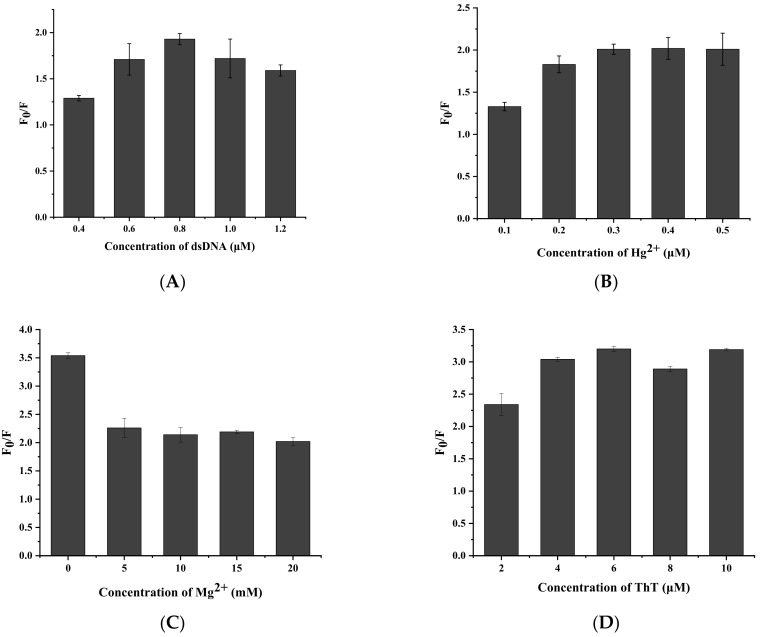
Optimization of GSH detection conditions: (**A**) dsDNA probe concentration; (**B**) Hg^2+^ concentration; (**C**) Mg^2+^ concentration; (**D**) ThT concentration. F and F0 represent the fluorescence intensities in the presence and absence of GSH, respectively.

In order to achieve better results in detecting GSH, which was based on the rule of low cost and high sensitivity, the following conditions were optimized: concentrations of dsDNA probe (0.4–1.2 μM), Hg^2+^ (0.1–0.5 μM), Mg^2+^ (0–20 mM) and ThT (2–10 μM). The relevant experimental parameters are shown in Figure 2, and the following conditions contributed the best results: a. 0.8 μM dsDNA; b. 0.4 μM Hg^2+^; c. 0 mM Mg^2+^; d. 6 μM ThT. Therefore, these chosen conditions were applied in the remaining experiments.

### 2.4. Quantification of GSH

**Figure 3:**
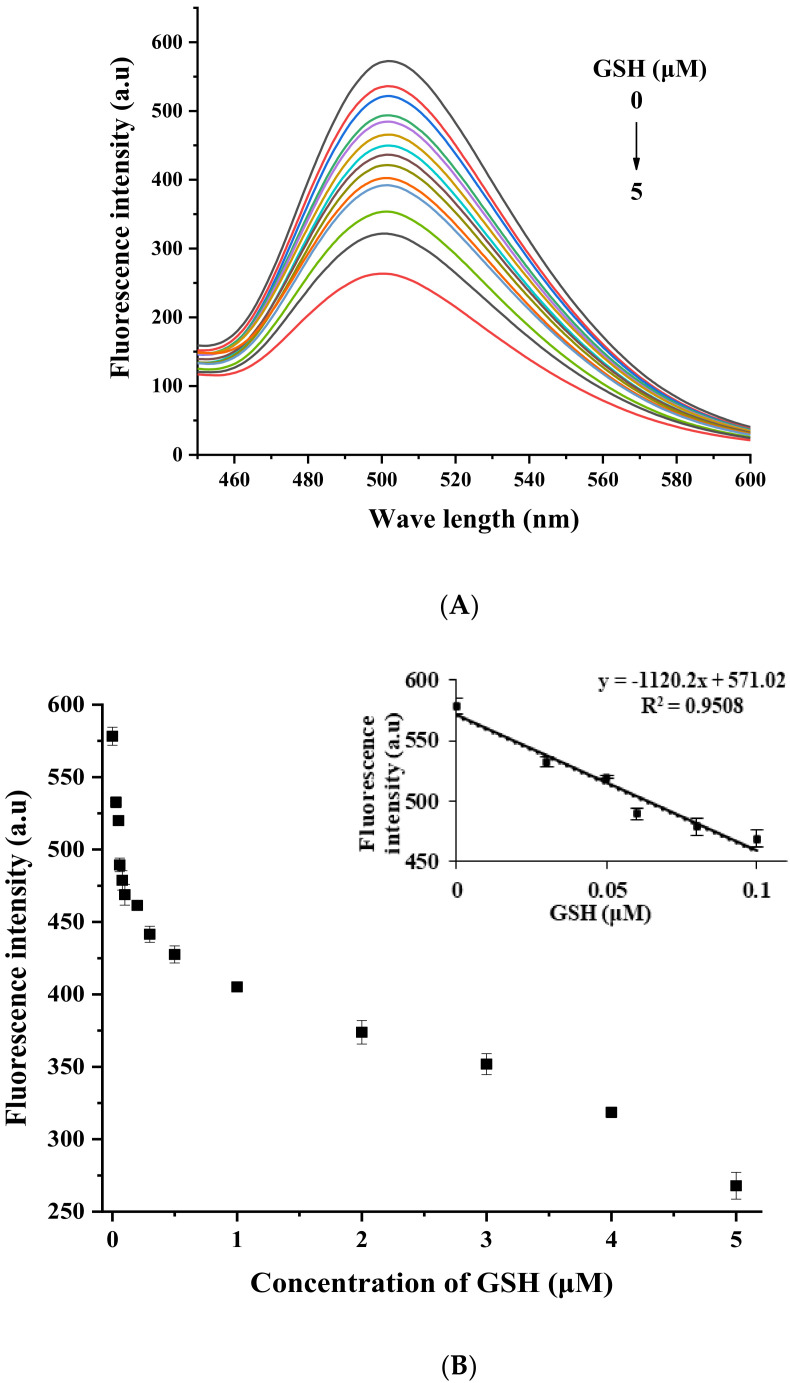
(**A**) Fluorescent signal response with varying concentrations of GSH. Arrow direction shows GSH concentration of 0, 0.03, 0.05, 0.06, 0.08, 0.1, 0.2, 0.3, 0.5, 1, 2, 3, 4, and 5 μM. (**B**) Fluorescence toward GSH concentration. Insert shows the linear relationship, which was plotted with fluorescence intensity vs. GSH concentration.

In order to evaluate the applicability of the proposed assay, by utilizing the selected experimental parameters, GSH detection was performed using a varying concentration of GSH (0, 0.03, 0.05, 0.06, 0.08, 0.1, 0.2, 0.3, 0.5, 1, 2, 3, 4, and 5 μM). The fluorescence intensity spectra are displayed in Figure 3A. These reflect that when GSH concentration was raised from 0 to 5 μM, the fluorescence intensity signal decreased rapidly. A correlation between the fluorescence intensity and concentration of GSH is shown in Figure 3B. Additionally, the insertion of Figure 3B displays the linear relationship (R^2^ = 0.9508) of GSH concentration in the range of 0–0.1 μM, which provides the regression equation Y = −1120.2 X + 571.02 (X is GSH concentration, μM). The calculated limit of detection (LOD) of this assay was 9.8 nM (3σ rule), which is similar to or superior to those of other assays (Table 1) [13,29,30,31,32,33].

### 2.5. Selectivity Study

**Figure 4:**
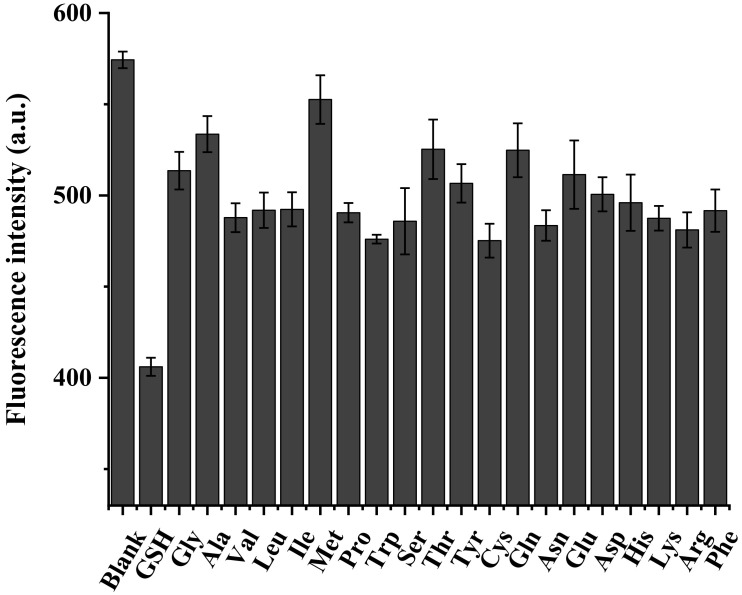
Selectivity of the proposed GSH towards interference by different amino acids. The concentrations of GSH and all amino acids were kept constant at 500 nM.

High selectivity is an important feature to evaluate a good sensor. In order to simulate complicated in vivo circumstances, and considering the similar structural features of amino acids, a variety of amino acids were employed to verify the selectivity of the proposed method. The concentrations of all amino acids and GSH were kept invariable at 500 nM. As shown in Figure 4, the emission spectra produced by the assessed amino acids were similar to blank, while a sharp reduction of fluorescence intensity was detected for GSH. Together, the distinct fluorescence results of amino acids demonstrated that the proposed method reveals excellent selectivity for the detection of GSH and is a hypothetical assay to be utilized in real samples.

### 2.6. GSH Assays in Biological Samples

To assess the present assay for practical applications, we operated the suggested GSH detection method in 1% human serum, which commonly contains biological substances. The related results are shown in Table 2. When we added 50, 100, and 200 nM of GSH into the diluted serum samples, the associated recovery rates were 96.54, 95.05 and 104.06%, respectively. The recovery rate of GSH in biological samples was between 90 and 110%, which demonstrates that the assay is reliable in the real samples. 

## 3. Materials and Methods

### 3.1. Reagents and Apparatus

The amino acids and DNA oligonucleotides used in this work were synthesized by Sangon Biotech (Shanghai) Co., Ltd. (Shanghai, China), and the sequence of DNA1 was 5’-TTTTTTTTT GTGGGT-3’, and that of DNA2 was 5’-GGGTGGGTGG TTATTTATT-3’ [27]. Thioflavin T (ThT), glutathione (GSH), and tris (hydroxymethyl) aminomethane (Tris) were obtained from Sigma-Aldrich (St. Louis. MO, USA). Hg(NO_3_)_2_ and Mg(NO_3_)_2_ were provided by Sinopharm Group CO. LTD. (Shanghai, China). All the chemicals used were analytical reagent grade and without further purification.

The fluorescence investigations were performed on an F-2700 fluorescence spectrophotometer (Hitachi, Japan) with excitation at a wavelength of 425 nm and emission at a wavelength of 450–600 nm. Both excitation and emission slits were set to 5 nm. In this study, each experiment was performed in the final solution with a volume of 100 μL. Three replicate measurements were included in each experiment, and the error bars in this paper were generated. Origin 2018 was adjusted to make it suitable for the experimental data.

### 3.2. Preparation of Solutions

DNA1 (100 μM) and DNA2 (100 μM) were dissolved in ultrapure water to make stock solutions, then heated to 95 °C for 10 min, and cooled down to 25 °C. Later, DNA1 and DNA2 with a concentration of 50 μM were mixed and incubated at 25 °C for 1 h and then frozen at −20 °C for future use [27]. The assay reaction buffer was Tris-HNO_3_ with a pH of 7.4 and a concentration of 10 mM.

### 3.3. Investigation of Feasibility

To explore the feasibility of the assay, we prepared three kinds of samples: A: DNA1/DNA2+Hg^2+^; B: DNA1/DNA2+Hg^2+^+GSH; C: DNA1/DNA2. In sample A, 0.8 μM dsDNA and 300 nM Hg^2+^ were added into Tris-HNO_3_ reaction buffer, but in sample B, 10 μM GSH, 0.8 μM dsDNA, and 0.3 μM Hg^2+^ were mixed, and in sample C, only 0.8 μM dsDNA was added into Tris-HNO3 reaction buffer. All of the mixture solutions were heated at 37 °C for 30 min. Finally, 6 μM of ThT was added into the mixture solutions, and the total volume of samples was brought to 100 μL. After 10 min, the fluorescence emission spectra were measured by F-2700. Three replicate measurements of each sample were performed.

### 3.4. Optimization of Analysis Conditions

To achieve maximum reaction efficacy and first-rate settings to execute the assay, we optimized some key reaction parameters, including various concentrations of dsDNA probe (0.4–1.2 μM), Hg^2+^ (0.1–0.5 μM), Mg^2+^ (0–20 mM), and ThT (2–10 μM).

### 3.5. Fluorescence Detection of GSH

In order to analyze GSH in the optimized conditions, we prepared thirteen samples for the subsequent experimentation. Different concentrations of GSH (0, 0.03, 0.05, 0.06, 0.08, 0.1, 0.2, 0.3, 0.5, 1, 2, 3, 4, and 5 μM) were mixed with 0.8 μM dsDNA and 0.4 μM Hg^2+^ into Tris-HNO_3_ reaction buffer and incubated at 37 °C for 30 min, followed by the addition of 6 μM ThT, and the final volume was set to 100 μL. The fluorescence was quantified after 10 min in a dark room.

For the detection of GSH in real samples, human serum was diluted 100 times with a Tris-HNO_3_ reaction buffer, and different concentrations of GSH were added to the Tris-HNO_3_ reaction buffer to prepare the spiked samples.

### 3.6. Selectivity

In order to determine the selectivity of the proposed method, we added a variety of amino acids, including Gly, Ala, Val, Leu, Ile, Met, Pro, Phe, Trp, Ser, Thr, Tyr, Cys, Gln, Asn, Glu, Asp, His, Lys, and Arg (500 nM), to a Tris-HNO_3_ reaction buffer that contained 0.8 μM dsDNA and 0.4 μM Hg^2+^ and incubated them for 30 min at 37 °C. After that, 6 μM of ThT was added, and a fluorescence measurement was taken after 10 min.

## 4. Conclusions

In summary, we established a rapid, facile, highly selective, and sensitive method for the detection of GSH based on a ThT/G-quadruplex system. According to the suggested method, Hg^2+^ interacts with a thymine–thymine mismatch to form T-Hg-T pairs to close the distance between DNA1 and DNA2 and directs two G-rich sides to construct a split G-quadruplex, resulting in interaction with ThT and significant escalation in fluorescent intensity. When GSH is introduced in the procedure, it can chelate Hg^2+^ to disrupt the structure of T-Hg-T mismatch pairs and interrupt the G-quadruplex formation. The proposed sensor could provide highly sensitive and selective detection of GSH in the linear range of 0–100 nM with an LOD of 9.8 nM, which is similar to or lower than that of other detection assays previously reported. Again, we employed a variety of amino acids to test the selectivity of the assay, and the outcome showed that the fluorescent intensity is similar to that of the blank samples, except for the group with added GSH, which means that the assay we established has high selectivity. Finally, this method was applied to human serum samples, which displayed a satisfactory recovery rate, indicating that the proposed assay has the potential to be utilized in real biological samples. Our study presents a strategy of GSH detection that is developed on the foundation of a well-known ThT/G-quadruplex system, and our findings suggest that it could become a useful tool for clinical diagnostics.

## Figures and Tables

**Table 1:** Comparison of different methods for detection of GSH.

Method	Probe	Linear Range	LOD	Reference
ECL	Ru(bpy)_2_(cpaphen)^2+^/TPrA/TiO_2_	5–215 μM	0.33 μM	[29]
ECL	Au-nanocluster	1–10,000 nM; 0.01–100 mM	0.32 nM	[30]
Colorimetry	Ultrathin MnO_2_ nanoflakes	2–200 μM	0.67 μM	[31]
Colorimetry	Silver nanoparticles	0.5–10 μM	0.368 μM	[32]
Fluorescence	ThT/G-quadruplex	30–2000 nM	13.9 nM	[13]
Fluorescence	MnO_2_ NS on BNQDs	0.5–250 μM	160 nM	[33]
Fluorescence	ThT/G-quadruplex	30–5000 nM	9.8 nM	This work

**Table 2:** Recovery experiments of GSH in diluted human serum using this method.

Sample Number	Added (nM)	Detected (nM)	Recovery (%)	R.S.D(%)
1	0	54.50 ± 4.08	-	6.66
2	50	102.77 ± 3.93	96.54	3.38
3	100	149.55 ± 6.43	95.05	3.89
4	200	262.62 ± 4.40	104.06	1.51

## Data Availability

The data presented in this study are available on request from the corresponding authors.

## Sample Availability

Samples are not available from the authors.
